# Prevalence of Myopia in Children Before, During, and After COVID-19 Restrictions in Hong Kong

**DOI:** 10.1001/jamanetworkopen.2023.4080

**Published:** 2023-03-22

**Authors:** Xiu Juan Zhang, Yuzhou Zhang, Ka Wai Kam, Fangyao Tang, Yi Li, Mandy P. H. Ng, Alvin L. Young, Patrick Ip, Clement C. Tham, Li Jia Chen, Chi Pui Pang, Jason C. Yam

**Affiliations:** 1 Department of Ophthalmology and Visual Sciences, The Chinese University of Hong Kong, Hong Kong SAR, China; 2 Joint Shantou International Eye Center of Shantou University and The Chinese University of Hong Kong, Shantou, China; 3 Department of Ophthalmology and Visual Sciences, Prince of Wales Hospital, Hong Kong SAR, China; 4 Department of Paediatrics and Adolescent Medicine, Li Ka Shing Faculty of Medicine, The University of Hong Kong, Hong Kong SAR, China; 5 Hong Kong Eye Hospital, Kowloon, Hong Kong SAR, China; 6 Department of Ophthalmology, Hong Kong Children’s Hospital, Hong Kong SAR, China; 7 Hong Kong Hub of Paediatric Excellence, The Chinese University of Hong Kong, Hong Kong SAR, China

## Abstract

**Importance:**

Childhood myopia increased during the COVID-19 pandemic. Limited evidence exists about whether myopia development was reversed or worsened after the lockdown.

**Objective:**

To determine the prevalence of myopia and its associated factors before, during, and after COVID-19 restrictions.

**Design, Setting, and Participants:**

This population-based, repeated cross-sectional study evaluated children aged 6 to 8 years from the Hong Kong Children Eye Study between 2015 and 2021 in 3 cohorts: before COVID-19 (2015-2019), during COVID-19 restrictions (2020), and after COVID-19 restrictions were lifted (2021).

**Exposures:**

All the children received ocular examinations, including cycloplegic autorefraction and axial length. Data about the children’s lifestyle, including time spent outdoors, near-work time, and screen time, were collected from a standardized questionnaire.

**Main Outcomes and Measures:**

The main outcomes were the prevalence of myopia, mean spherical equivalent refraction, axial length, changes in lifestyle, and the associated factors over 7 years. Data were analyzed using descriptive statistics, logistic regression, and generalized estimating equations.

**Results:**

Of 20 527 children (mean [SD] age, 7.33 [0.89] years; 52.8% boys and 47.2% girls), myopia prevalence was stable from 2015 to 2019 (23.5%-24.9%; *P* = .90) but increased to 28.8% (*P* < .001) in 2020 and 36.2% (*P* < .001) in 2021. The mean (SD) time spent outdoors was much lower in 2020 (0.85 [0.53] h/d; *P* < .001) and 2021 (1.26 [0.48] h/d; *P* < .001) compared with pre–COVID-19 levels (1.40 [0.47]-1.46 [0.65] h/d). The trend was reversed for total near-work time and screen time. High myopia prevalence was associated with the COVID-19 pandemic (odds ratio [OR], 1.40; 95% CI, 1.28-1.54; *P* < .001), younger age (OR, 1.84; 95% CI, 1.76-1.93; *P* < .001), male sex (OR, 1.11; 95% CI, 1.03-1.21; *P* = .007), lower family income (OR, 1.05; 95% CI, 1.00-1.09; *P* = .04), and parental myopia (OR, 1.61; 95% CI, 1.52-1.70; *P* < .001). During the pandemic, mean (SD) near-work and screen times in children from lower-income families were 5.16 (2.05) h/d and 3.44 (1.97) h/d, more than from higher-income families (4.83 [1.85] and 2.90 [1.61] h/d, respectively).

**Conclusions and Relevance:**

The findings of this cross-sectional study revealed that after COVID-19 restrictions were lifted in Hong Kong, myopia prevalence among children was higher than before the pandemic, and lifestyle did not return to pre–COVID-19 levels. Younger children and those from low-income families were at a higher risk of myopia development during the pandemic, suggesting that collective efforts for myopia control should be advocated for these groups.

## Key Points

**Question:**

Was myopia development in children reversed or worsened after the COVID-19 lockdown in Hong Kong?

**Findings:**

In this cross-sectional study of 20 587 children, after COVID-19 restrictions were lifted, myopia prevalence was still high, and time spent outdoors, near-work time, and screen time did not return to pre–COVID-19 levels. Younger children and children from low-income families were at a higher risk of myopia during the pandemic.

**Meaning:**

The study’s findings suggested that increasing time spent outdoors and reducing screen and near-work time may prevent burgeoning postpandemic myopia, particularly among younger children and children from low-income families.

## Teaser

This cross-sectional study evaluates the prevalence of myopia and its associated factors in children before, during, and after the COVID-19 pandemic in Hong Kong.

## Introduction

Myopia has emerged as a major public health concern worldwide, especially in East and Southeast Asia.^1,2,3^ It is estimated that approximately one-half of the world’s population will become myopic by the year 2050 and that one-tenth will become highly myopic.^1^ Myopia is a major cause of visual disability in children, and children with myopia are predisposed to multiple ocular complications, thereby increasing the risk of irreversible vision loss later in life.^4^

As a result of the COVID-19 global pandemic, there has been a dramatic change in lifestyle and behavior in children, including reduced time outdoors and increased electronic learning onscreen.^5^ Recent reports have shown that myopia increased in children at the early stage of the COVID-19 pandemic, when restriction measures were imposed to prevent the spread of the virus.^6,7,8,9^ With burgeoning vaccination and the epidemic under control, lockdown measures were withdrawn in many countries and cities. Currently, there is limited evidence about whether myopia development was reversed or worsened after the lockdown.^10^ The underlying causes of the changes in myopia occurrence as associated with the COVID-19 pandemic are still under investigation.

Using cycloplegic refraction and axial length (AL), we conducted a repeated cross-sectional study from 2015 to 2021 to investigate the trend of myopia prevalence, lifestyle changes, and the associated factors in schoolchildren in Hong Kong. Based on the quarantine policy of the Government of Hong Kong and the Education Bureau,^11^ these 7 consecutive years included the period before, during, and after COVID-19 restrictions for schoolchildren. The study aimed to answer the following questions: (1) What is the myopia prevalence after relaxing restriction measures? (2) Did lifestyle, including time spent outdoors, near-work time, and screen time, change after restrictions were lifted? (3) Were there any risk factors associated with myopia during this period?

## Methods

### Study Population

Participants for this cross-sectional study were recruited from the ongoing Hong Kong Children Eye Study (HKCES), a population-based study of eye conditions among schoolchildren aged 6 to 8 years.^2,12,13,14,15,16,17,18^ The sample selection was based on a stratified and clustered randomized sampling frame. We stratified all 571 primary schools registered with the Education Bureau into the 7 cluster regions used by the Hospital Authority Services in Hong Kong. The primary schools in each cluster region were randomly assigned an invitation priority according to the ranking numbers generated by computer in each year. The students from grades 1 to 3 (ie, aged 6-8 years) in each selected school were recruited. In terms of sample calculation for annual myopia prevalence, based on previous reports in Hong Kong,^19^ we assumed myopia prevalence in children aged 6 to 8 years to be 26.4% and the absolute error to be 2%; thus, a sample size of 953 in each year would be needed to achieve 80% power at a .05 significance level.

All children were invited annually to the The Chinese University of Hong Kong Eye Centre for comprehensive ocular examinations and to answer standardized questionnaires according to the unified protocol.^2^ The study protocol was approved by the ethics committee board of The Chinese University of Hong Kong. All children and their parents signed a written informed consent before their participation in the study. All study procedures adhered to the tenets of the Declaration of Helsinki.^20^ The study followed the Strengthening the Reporting of Observational Studies in Epidemiology (STROBE) reporting guideline.

At the beginning of the COVID-19 pandemic in January 2020, the Government of Hong Kong ordered school closures^11^ and initiated subsequent online classes instead of in-person learning. Face-to-face classes resumed for all schools in March 2021. There was no home confinement or additional digital learning in the afternoon sections. COVID-19 restrictions for children were defined as the period of school closure with quarantine measures. Different participants from 2015 to 2021 were included during 3 distinct periods: before the COVID-19 pandemic from 2015 to December 2019, during COVID-19 restrictions in 2020, and after COVID-19 restrictions from March 2021 onward.

### Ocular Examinations

Visual acuity was assessed using a logarithm of the minimum angle of resolution chart (NIDEK Co, Ltd). Cycloplegic autorefraction was performed using an autorefractor (ARK-510A; NIDEK Co, Ltd) after a cycloplegic regimen, which consisted of at least 2 cycles of eye drops. In the first cycle, 2 separate eye drops, cyclopentolate 1% (Cyclogyl; Alcon-Couvreur) and tropicamide 1% (Santen Pharmaceutical Co, Ltd), were administered to both eyes at 5 minutes apart. The second cycle of the same cycloplegic eye drops was administered 10 minutes after the first. Ocular axial length (AL) was measured with partial coherence laser interferometry (IOLMaster 700; Carl Zeiss Meditec).

### Questionnaires on Children’s Outdoor Activities, Screen Time, and Near-Work Time

Validated questionnaires used in this study were derived mainly from the Chinese version of questionnaires used in the Sydney Myopia Study^21^ and adapted in the HKCES.^2^ Total outdoor time was divided into 2 categories: leisure activity and sports. Reading and writing time were defined as time for any near-visual activities, excluding time engaged with electronic devices. Screen time was defined as time spent using a computer and any handheld electronic devices. Total near work was defined as the sum of total reading and writing time and screen time, excluding watching television and videos. All the variables were collected separately for weekdays and weekends. The average number of hours per day was calculated using the following equation: {[(weekday hours spent) × 5] + [(weekend hours spent) × 2]}/7. Watching television, videos, and compact discs were classified as midrange activities. Diopter-hour was calculated using the following equation: [(hours spent studying + hours spent reading for pleasure) × 3] + [(hours spent playing video games or working on the computer at home) × 2] + [(hours spent watching television) × 1].^22,23^ Parental myopia was defined as 1 or both parents having myopia (details provided in the eMethods in Supplement 1). Family income was defined as the income shared by people living in the same household. Using the median family monthly income in Hong Kong of HK$26 300 (approximately US $3400) as a reference,^24^ a family income of HK$25 000 (approximately US $3200) was designated as a cutoff, with families earning less defined as lower-income families.

### Definition and Outcomes

Spherical equivalent refraction (SER) was defined as spherical diopters plus one-half cylindrical diopters. Myopia was defined as an SER of −0.50 diopters (D) or less. The outcomes were (1) prevalence of myopia, mean SER, and AL over the 7 years studied; (2) time spent outdoors, near-work time, and screen time during the period; and (3) associated factors, including age, sex, time spent on outdoor activities, near-work time, screen time, diopter-hours, family income, and parental myopia. The COVID-19 pandemic was defined as the period from January 24, 2020, onward.

### Statistical Analysis

The demographic characteristics of participants were summarized using descriptive statistics. Continuous variables were reported in terms of means and SDs, while categorical variables were reported in terms of counts and percentages. Group differences in data were tested using Student *t* test for continuous variables and Pearson χ^2^ test for categorical variables. For the participant-based outcome (myopia), we used logistic regression and for the eye-based outcomes (SER, AL), generalized estimating equations to calculate the association between risk factors of myopia and the interactions of myopia with the COVID-19 pandemic. Analyses of the association between the COVID-19 pandemic and age, sex, parental myopia, and family income with SER and AL were based on data included for both eyes, using generalized estimating equations to adjust for intereye correlation within the same participant. All statistical analyses were performed using SPSS, version 24.0 software (IBM). Two-sided *P* < .05 was considered statistically significant.

## Results

### Study Population

A total of 20 527 children (41 054 eyes; boys, 10 828 [52.8%]; girls, 9699 [47.2%]; mean [SD] age, 7.33 [0.89] years) were included in this study. The sex ratio, mean age, family income, and parental myopia were not significantly different across the 7 years studied (Table 1).

**Table 1.** Participant Demographic Characteristics

	2015 (n = 1037)	2016 (n = 1536)	2017 (n = 1370)	2018 (n = 5516)	2019 (n = 7127)	2020 (n = 1211)	2021 (n = 2730)	Total (n = 20 527)
Age, mean (SD), y	7.47 (0.77)	7.37 (0.85)	7.50 (0.83)	7.19 (0.96)	7.25 (0.89)	7.34 (0.78)	7.60 (0.84)	7.33 (0.89)
Sex, No. (%)								
Male	513 (49.5)	787 (51.2)	747 (54.5)	2961 (53.7)	3752 (52.6)	617 (50.9)	1451 (53.2)	10 828 (52.8)
Female	524 (50.5)	749 (48.8)	623 (45.5)	2555 (46.3)	3375 (47.4)	594 (49.1)	1279 (46.8)	9699 (47.2)
Family income less than HK$25 000/mo, No. (%)^a^	402 (38.8)	634 (41.3)	578 (42.2)	2361 (42.8)	2715 (38.1)	459 (37.9)	1103 (40.4)	8252 (40.2)
Parents with myopia in <2 eyes, No. (%)	661 (63.7)	1000 (65.1)	908 (66.3)	3712 (67.3)	5124 (71.9)	804 (66.4)	1906 (69.8)	14 115 (68.8)

^a^ HK$25 000 is equivalent to US $3184.61 as of February 14, 2023.

### Prevalence of Myopia, SER, and AL

The prevalence of myopia was stable from 2015 to 2019 (23.5%-24.9%; *P* = .90) but increased to 28.8% (*P* < .001) in 2020 and 36.2% (*P* < .001) in 2021. In 2020 and 2021, significantly increased myopia prevalence was observed in children aged 6 years (18.5% [*P* = .01] and 25.2% [*P* < .001]) and 7 years (30.4% [*P* = .02] and 34.1% [*P* < .001]) but not in children aged 8 years in 2020 (42.1% [*P* = .13] vs 46.0% in 2021 [*P* < .001]) (Table 2 and Figure).

**Table 2.** Annual Myopia Prevalence, Spherical Equivalent Refraction, and Axial Length by Age and Sex, 2015-2021

	No.	(1) Before COVID-19 pandemic					*P* value	(2) During COVID-19 restriction, 2020	(3) After COVID-19 restriction, 2021	*P* value^b^	
		2015	2016	2017	2018	2019				(2) vs (1)	(3) vs (1)
**Myopia**^a^ **prevalence, No. (%)**											
Total	20 527	259 (24.9)	363 (23.6)	326 (23.8)	1295 (23.5)	1696(23.8)	.90	349 (28.8)	987 (36.2)	<.001	<.001
Age, y											
6	7993	39 (12.7)	71 (12.7)	60 (13.7)	338 (13.6)	445 (14.4)	.76	74 (18.5)	181 (25.2)	.01	<.001
7	7090	117 (25.8)	147 (26.4)	125 (24.7)	435 (26.2)	601 (25.6)	.95	174 (30.4)	340 (34.1)	.02	<.001
8	5444	103 (37.2)	145 (34.5)	141 (33.2)	522 (37.9)	650 (38.4)	.23	101 (42.1)	466 (46.0)	.13	<.001
Sex											
Male	10 828	126 (24.6)	203 (25.8)	169 (22.6)	687 (23.2)	914 (24.4)	.50	175 (28.4)	544 (37.5)	.01	<.001
Female	9699	133 (25.4)	160 (21.4)	157 (25.2)	608 (23.8)	782 (23.2)	.49	174 (29.3)	443 (34.7)	.001	<.001
**Spherical equivalent refraction, mean (SD), diopters^c^**											
Total	20 527	0.23 (1.29)	0.25 (1.43)	0.25 (1.38)	0.30 (1.47)	0.27 (1.51)	.46	0.11 (1.34)	−0.08 (1.57)	.001	<.001
Age, y											
6	7993	0.62 (0.97)	0.65 (1.24)	0.67 (1.11)	0.66 (1.20)	0.61 (1.24)	.70	0.43 (1.11)	0.30 (1.48)	.01	<.001
7	7090	0.24 (1.23)	0.20 (1.41)	0.25 (1.31)	0.23 (1.46)	0.19 (1.59)	.89	0.08 (1.31)	−0.03 (1.47)	.03	<.001
8	5444	−0.22 (1.55)	−0.19 (1.54)	−0.21 (1.58)	−0.24 (1.72)	−0.23 (1.69)	.99	−0.34 (1.59)	−0.39 (1.66)	.29	.01
Sex											
Male	10 828	0.22 (1.30)	0.23 (1.41)	0.24 (1.41)	0.29 (1.50)	0.24 (1.53)	.78	0.14 (1.29)	−0.17 (1.57)	.04	<.001
Female	9699	0.23 (1.29)	0.28 (1.43)	0.26 (1.35)	0.32 (1.43)	0.31 (1.49)	.64	0.08 (1.39)	0.03 (1.56)	<.001	<.001
**Axial length, mean (SD), mm^c^**											
Total	20 527	23.04 (0.86)	23.04 (0.89)	23.05 (0.89)	22.99 (0.89)	22.99 (0.92)	.68	23.07 (0.85)	23.21 (0.96)	.03	<.001
Age, y											
6	7993	22.73 (0.68)	22.74 (0.74)	22.73 (0.72)	22.69 (0.77)	22.71 (0.81)	.87	22.78 (0.80)	22.85 (0.94)	.19	<.001
7	7090	23.05 (0.90)	23.08 (0.92)	23.06 (0.87)	23.09 (0.89)	23.08 (0.90)	.89	23.12 (0.82)	23.20 (0.90)	.18	.001
8	5444	23.37 (0.85)	23.38 (0.89)	23.37 (0.95)	23.39 (0.91)	23.37 (0.95)	.92	23.43 (0.85)	23.47 (0.96)	.34	.01
Sex											
Male	10 828	23.22 (0.81)	23.23 (0.83)	23.28 (0.86)	23.22 (0.87)	23.26 (0.89)	.23	23.33 (0.81)	23.49 (0.93)	.15	<.001
Female	9699	22.68 (0.82)	22.74 (0.85)	22.68 (0.84)	22.72 (0.84)	22.70 (0.86)	.83	22.80 (0.81)	22.89 (0.90)	.11	.002

^a^ Myopia was defined as spherical equivalent refraction less than or equal to −0.5 diopters in at least 1 eye.

^b^ *P* values were generated by generalized estimating equations adjusted for age and sex.

^c^ Mean and SD were calculated using data from both eyes.

**Figure.**
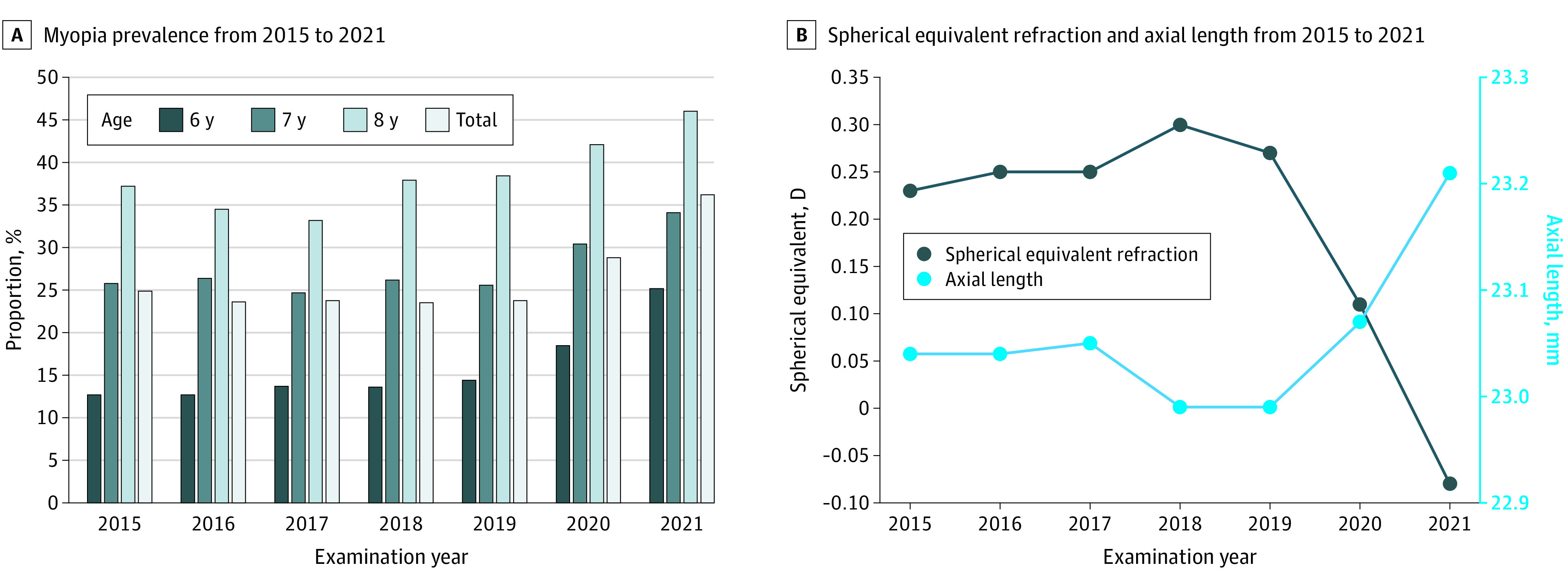
Annual Myopia Prevalence, Spherical Equivalent Refraction, and Axial Length, 2015-2021

The mean (SD) SER was stable (0.23 [1.29]-0.30 [1.47] D; *P* = .46) from 2015 to 2019 and decreased in 2020 (0.11 [1.34] D; *P* = .001) and 2021 (−0.08 [1.57] D; *P* < .001). Similar trends were observed in AL changes for all age and both sex groups (Table 2 and Figure).

### Outdoor Time, Near-Work Time, Screen Time, and Diopter-Hours

The mean (SD) time spent outdoors was 1.40 (0.47) to 1.46 (0.65) hours per day from 2015 to 2019. It decreased to 0.85 (0.53) hours per day in 2020 (*P* < .001) and returned to 1.26 (0.48) hours per day in 2021, which was still lower than the years before 2019 (*P* < .001). Similarly, the mean (SD) near-work time, screen time, and diopter-hours were 3.23 (1.31) to 3.49 (1.50) hours per day, 1.93 (1.24) to 2.09 (1.28) hours per day, and 9.54 (3.59) to 10.28 (4.18) hours per day, respectively, from 2015 to 2019, increased to 5.72 (1.61), 3.56 (1.50), and 16.36 (4.37) hours per day in 2020 (*P* < .001), and returned to 4.64 (1.90), 2.96 (1.78), and 13.41 (5.01) hours per day, which was still higher than the years before 2019 (*P* < .001). During and after the pandemic restrictions, boys spent more time on computers and electronic devices (mean [SD], 3.65 [1.67] and 3.01 [1.93] hours per day) than girls (mean [SD], 3.46 [1.26] and 2.90 [1.73] hours per day) (Table 3).

**Table 3.** Annual Time Spent Outdoors, Near-Work Time, Screen Time, and Diopter-Hours, 2015-2021

	No.	Mean (SD) hours per day							*P* value^a^	
		(1) Before COVID-19 pandemic					(2) During COVID-19 restriction, 2020	(3) After COVID-19 restriction, 2021	(2) vs (1)	(3) vs (1)
		2015	2016	2017	2018	2019				
**Time spent outdoors**										
Total	20 527	1.40 (0.47)	1.42 (0.68)	1.45 (0.59)	1.41 (0.59)	1.46 (0.65)	0.85 (0.53)	1.26 (0.48)	<.001	<.001
Age, y										
6	7993	1.40 (0.46)	1.43 (0.65)	1.45 (0.58)	1.43 (0.60)	1.50 (0.67)	0.83 (0.56)	1.26 (0.49)	<.001	<.001
7	7090	1.40 (0.49)	1.44 (0.71)	1.48 (0.60)	1.41 (0.58)	1.44 (0.61)	0.89 (0.53)	1.25 (0.45)	<.001	<.001
8	5444	1.40 (0.45)	1.39 (0.66)	1.41 (0.57)	1.40 (0.58)	1.43 (0.64)	0.79 (0.52)	1.29 (0.51)	<.001	<.001
Sex										
Male	10 828	1.43 (0.47)	1.46 (0.70)	1.49 (0.61)	1.45 (0.59)	1.50 (0.64)	0.88 (0.52)	1.28 (0.48)	<.001	<.001
Female	9699	1.37 (0.47)	1.39 (0.65)	1.39 (0.55)	1.37 (0.59)	1.42 (0.65)	0.83 (0.55)	1.25 (0.49)	<.001	<.001
**Near-work time**										
Total	20 527	3.49 (1.50)	3.47 (1.39)	3.47 (1.27)	3.23 (1.31)	3.30 (1.38)	5.72 (1.61)	4.64 (1.90)	<.001	<.001
Age, y										
6	7993	3.34 (1.38)	3.20 (1.32)	3.27 (1.27)	3.01 (1.21)	3.11 (1.30)	5.40 (1.33)	4.53 (1.99)	<.001	<.001
7	7090	3.53 (1.65)	3.56 (1.24)	3.56 (1.28)	3.33 (1.35)	3.37 (1.39)	5.67 (1.51)	4.59 (1.80)	<.001	<.001
8	5444	3.62 (1.33)	3.71 (1.59)	3.55 (1.24)	3.54 (1.36)	3.57 (1.46)	6.40 (2.10)	4.75 (1.94)	<.001	<.001
Sex										
Male	10 828	3.49 (1.59)	3.49 (1.46)	3.51 (1.23)	3.26 (1.26)	3.34 (1.41)	5.72 (1.46)	4.72 (1.91)	<.001	<.001
Female	9699	3.49 (1.40)	3.45 (1.33)	3.43 (1.30)	3.21 (1.35)	3.27 (1.36)	5.71 (1.73)	4.56 (1.89)	<.001	<.001
**Screen time**										
Total	20 527	1.93 (1.24)	1.98 (1.16)	2.08 (1.09)	2.02 (1.21)	2.09 (1.28)	3.56 (1.50)	2.96 (1.78)	<.001	<.001
Age, y										
6	7993	1.89 (1.21)	1.81 (1.07)	1.91 (1.08)	1.90 (1.19)	2.01 (1.23)	3.28 (1.15)	2.95 (1.90)	<.001	<.001
7	7090	1.94 (1.36)	2.05 (1.15)	2.12 (1.06)	1.99 (1.14)	2.06 (1.21)	3.45 (1.36)	2.90 (1.67)	<.001	<.001
8	5444	1.95 (1.01)	2.10 (1.25)	2.20 (1.13)	2.27 (1.29)	2.27 (1.44)	4.42 (2.03)	3.02 (1.81)	<.001	<.001
Sex										
Male	10 828	1.95 (1.16)	1.98 (1.07)	2.08 (1.11)	2.07 (1.25)	2.15 (1.31)	3.65 (1.67)	3.01 (1.83)	<.001	<.001
Female	9699	1.91 (1.31)	1.97 (1.25)	2.07 (1.08)	1.95 (1.16)	2.02 (1.25)	3.46 (1.26)	2.90 (1.73)	<.001	<.001
**Diopter-hours**										
Total	20 527	10.28 (4.18)	10.14 (3.84)	10.18 (3.55)	9.54 (3.59)	9.77 (3.84)	16.36 (4.37)	13.41 (5.01)	<.001	<.001
Age, y										
6	7993	9.88 (3.86)	9.44 (3.68)	9.61 (3.52)	8.95 (3.28)	9.27 (3.63)	15.51 (3.74)	13.09 (5.14)	<.001	<.001
7	7090	10.39 (4.54)	10.39 (3.49)	10.47 (3.48)	9.79 (3.77)	9.95 (3.90)	16.22 (4.13)	13.31 (4.75)	<.001	<.001
8	5444	10.57 (3.80)	10.75 (4.33)	10.41 (3.59)	10.32 (3.73)	10.46 (4.01)	18.17 (5.49)	13.72 (5.18)	<.001	<.001
Sex										
Male	10 828	10.29 (3.95)	10.05 (3.73)	10.04 (3.63)	9.42 (3.66)	9.61 (3.75)	16.30 (4.63)	13.15 (4.91)	<.001	<.001
Female	9699	10.27 (4.39)	10.24 (3.97)	10.35 (3.44)	9.67 (3.50)	9.95 (3.93)	16.42 (4.05)	13.71 (5.12)	<.001	<.001

^a^ *P* values were generated by generalized estimating equations adjusted for age and sex.

### Association of COVID-19 Pandemic With Myopia

Among all 20 527 children, multivariable analysis showed that high myopia prevalence was associated with the COVID-19 pandemic (odds ratio [OR], 1.40; 95% CI, 1.28-1.54; *P* < .001), younger age (OR, 1.84; 95% CI, 1.76-1.93; *P* < .001), male sex (OR, 1.11; 95% CI, 1.03-1.21; *P* = .007), lower family income (OR, 1.05; 95% CI, 1.00-1.09; *P* = .04), and parental myopia (OR, 1.61; 95% CI, 1.52-1.70; *P* < .001) (Table 4). Similar associations were observed in myopic SER and AL changes (Table 4). Similar findings were observed in myopia prevalence when including time spent outdoors, near-work time, screen time, or diopter-hours, instead of the COVID-19 pandemic, in the models (eTable 1 in Supplement 1).

**Table 4.** Association Between COVID-19 Pandemic^a^ and Myopia Prevalence, Spherical Equivalent Refraction, and Axial Length

	Model 1		Model 2		Model 3		Model 4	
	OR (95% CI)	*P* value^b^	OR (95% CI)	*P* value^b^	OR (95% CI)	*P* value^b^	OR (95% CI)	*P* value^b^
**Myopia prevalence (no myopia as reference group)** ^c^								
Examination during COVID-19 pandemic, no as reference	1.58 (1.45 to 1.72)	<.001	1.40 (1.28 to 1.54)	<.001	1.78 (1.52 to 2.09)	<.001	NA	NA
Age	NA	NA	1.84 (1.76 to 1.93)	<.001	1.84 (1.76 to 1.93)	<.001	NA	NA
Sex, female as reference	NA	NA	1.11 (1.03 to 1.21)	.007	1.12 (1.03 to 1.21)	.006	NA	NA
Family income	NA	NA	1.05 (1.00 to 1.09)	.04	1.05 (1.00 to 1.09)	.03	NA	NA
No. of parental myopia	NA	NA	1.61 (1.52 to 1.70)	<.001	1.70 (1.59 to 1.80)	<.001	NA	NA
Examination during COVID-19 pandemic × parental myopia	NA	NA	NA	NA	0.81 (0.72 to 0.91)	<.001	NA	NA
**Spherical equivalent refraction** ^d^ ^,^ ^e^ ^,^ ^f^								
Examination during COVID-19 pandemic, no as reference	−0.29 (−0.36 to −0.23)	<.001	−0.20 (−0.26 to −014)	<.001	−0.30 (−0.40 to −0.21)	<.001	−0.38 (−0.58 to −0.19)	<.001
Age	NA	NA	−0.40 (−0.43 to −0.37)	<.001	−0.40 (−0.43 to −0.37)	<.001	−0.40 (−0.43 to −0.37)	<.001
Sex, female as reference	NA	NA	−0.10 (−0.15 to −0.05)	<.001	−0.10 (−0.15 to −0.06)	<.001	−0.10 (−0.15 to −0.06)	<.001
Family income	NA	NA	−0.06 (−0.09 to −0.04)	<.001	−0.06 (−0.09 to −0.04)	<.001	−0.05 (−0.08 to −0.02)	.001
No. of parental myopia	NA	NA	−0.35 (−0.38 to −0.31)	<.001	−0.37 (−0.40 to −0.33)	<.001	−0.35 (−0.38 to −0.31)	<.001
Examination during COVID-19 pandemic × parental myopia	NA	NA	NA	NA	0.10 (0.02 to 0.18)	.01	NA	NA
Examination during COVID-19 pandemic × family income	NA	NA	NA	NA	NA	NA	0.06 (0.00 to 0.13)	.04
**Axial length** ^d^ ^,^ ^e^ ^,^ ^f^								
Examination during COVID-19 pandemic, no as reference	0.16 (0.13 to 0.20)	<.001	0.07 (0.03 to 0.11)	<.001	0.12 (0.06 to 0.18)	<.001	NA	NA
Age	NA	NA	0.32 (0.31 to 0.34)	<.001	0.32 (0.31 to 0.34)	<.001	NA	NA
Sex, female as reference	NA	NA	0.55 (0.52 to 0.58)	<.001	0.55 (0.52 to 0.58)	<.001	NA	NA
Family income	NA	NA	0.02 (0.00 to 0.03)	.03	0.02 (0.00 to 0.03)	.03	NA	NA
No. of parental myopia	NA	NA	0.13 (0.12 to 0.15)	<.001	0.14 (0.12 to 0.16)	<.001	NA	NA
Examination during COVID-19 pandemic x parental myopia	NA	NA	NA	NA	−0.05 (−0.09 to 0.00)	.04	NA	NA

Abbreviation: NA, not applicable.

^a^ COVID-19 pandemic was defined as the period from January 24, 2020, to December 31, 2021.

^b^ *P* values generated by logistic regression. Time spent outdoors, near-work time, and screen time were not included in the models due to high correlation with the COVID-19 pandemic variable.

^c^ Myopia was defined as spherical equivalent refraction less than or equal to −0.5 diopters in at least 1 eye.

^d^ Data are β (95% CI) for spherical equivalent refraction and axial length.

^e^ Both eyes were used in the analyses for spherical equivalent refraction and axial length.

^f^ *P* values generated by the generalized estimating equation. Time spent outdoors, near-work time, and screen time were not included in the models due to high correlation with the COVID-19 pandemic variable.

There was significant interaction between the COVID-19 pandemic and parental myopia on myopia prevalence in children, showing that the less the parental myopia, the greater the influence of the pandemic childhood myopia prevalence (OR, 0.81; 95% CI, 0.72-0.91; *P* < .001). Similarly, there were significant interactions between the pandemic, parental myopia, and family income on SER changes, showing that the less the parental myopia or the lower the family income, the greater the influence of the pandemic on SER changes (β [95% CI], 0.10 [0.02-0.18; *P* = .01] and 0.06 [0.00-0.13; *P* = .04], respectively) (Table 4).

### Myopia and Lifestyle Changes Before and During the COVID-19 Pandemic in Different Subgroups

Subgroup analysis showed significant differences in myopia prevalence, SER, and AL before and during the COVID-19 pandemic, except among children whose parents had myopia in both eyes or whose family income amounted to HK$50 000 or more (eTable 2 in Supplement 1). During the COVID-19 pandemic, mean (SD) near-work time and screen time were, respectively, 5.16 (2.05) and 3.44 (1.97) hours per day for children living in households with incomes less than HK$25 000, which was higher than 4.83 (1.85) and 2.90 (1.61) hours per day for household incomes of HK$50 000 or more (eTable 3 in Supplement 1). Higher myopia prevalence was associated with less outdoor time (OR, 0.88; 95% CI, 0.78-0.99; *P* = .04), more near-work time (OR, 1.05; 95% CI, 1.00-1.09; *P* = .04), and more diopter-hours (OR, 1.02; 95% CI, 1.00-1.04; *P* = .03) in children from lower-income families (less than HK$25 000 per month). The association of myopia with near-work time and diopter-hours disappeared in families with higher incomes (eTable 4 in Supplement 1). Similar findings were observed in children with parents without myopia (eTable 5 in Supplement 1).

## Discussion

This cross-sectional study delineates the trend of myopia during 7 consecutive years that include the period before, during, and after the COVID-19 pandemic and restriction measures in Hong Kong. The prevalence of myopia increased not only during the COVID-19 restrictions but also after the restrictions were eased, at approximately 1.5-fold. The myopia prevalence was doubled in children aged 6 years after the pandemic. Lifestyle, including time spent outdoors, near-work time, and screen time, changed during the restriction period and did not return to pre–COVID-19 levels after the restrictions were lifted. Younger children, as well as those from families with a low household income, were at higher risk of myopia development during the pandemic.

### High Myopia Prevalence After COVID-19 Restrictions

Before the COVID-19 pandemic, Hong Kong had the highest prevalence of childhood myopia (25% for children aged 6-8 years) in the world.^2^ Consistent with reports suggesting that myopia worsened after the COVID-19 pandemic,^6,7,8,10^ we found a higher prevalence during the pandemic. Our results also showed that myopia prevalence was high even after restrictions were lifted. In addition, we observed that myopia was more severe in younger children during the COVID-19 pandemic. Myopia prevalence in children aged 6 years was approximately 13% from 2015 to 2019 in Hong Kong, much higher than the 0.2% to 7.4% reported in other regions of China^25,26,27,28,29,30,31,32^ and 6.6% in Singapore.^33^ During the COVID-19 pandemic, the prevalence nearly doubled (25% in 2021) among 6-year-old children. Studies in China^10^ and Korea^34^ also reported that younger children were more sensitive to lockdown with regard to myopic progression. The underlying reason remains to be investigated. Our study revealed more myopic changes in SER and AL after COVID-19 restrictions were lifted. However, a study in China reported that myopic progression was partially reversed after lockdown.^10^ This discrepancy may be explained by different refraction measurements. A strict cycloplegia protocol was used in our study to measure SER, thus eliminating accommodative spasm or pseudomyopia due to long near-work time during home confinement. Different school closure policies and home quarantine time might be another explanation. Nevertheless, our study’s findings suggest that the prevalence of myopia may remain high over the next few years.

### Associated Factors of Myopia During the COVID-19 Pandemic

Both increased near-work time and decreased time spent outdoors have been implicated in the development of myopia.^35^ Another important finding from our study is that the children’s lifestyle, including reduced time spent outdoors, increased near-work time, and increased screen time, did not completely return to pre–COVID-19 levels after the restrictions were lifted. During school closures, classrooms moved to digital platforms, compelling children to spend more time on digital devices and, therefore, increase total near-work time. Our study findings suggest that such behavior changes may persist beyond the pandemic.

Parental myopia is a known risk factor for childhood myopia development, suggesting a genetic contribution.^36^ Zadnik et al^23^ found that a history of parental myopia is associated with children’s ocular size. Consistently, our study findings also showed higher myopia prevalence and myopic SER among children with more parental myopia both before COVID-19 and during the pandemic (eTable 2 in Supplement 1). However, there was a larger worsening in myopia prevalence and SER changes during COVID-19 compared with before COVID-19 in children with less parental myopia, suggesting a higher risk for myopia development and myopic shift during the pandemic. Because parental myopia is one of the strongest factors associated with childhood myopia,^37^ for children with more parental myopia, the association of environmental changes, such as time spent outdoors and near-work time, during COVID-19 would be relatively weaker. On the other hand, for children with both parents without myopia, the association of parental myopia is minimal, and, therefore, the association of the change in environmental factors would be relatively stronger.

The evidence for socioeconomic background contributing to childhood myopia development is inconclusive. We found that children from low-income families were at a higher risk of myopic shift during the pandemic. The higher the family income, the less change in myopic SER and AL (Table 4). One possible explanation is that children from families with lower household incomes had longer near-work and screen times during the COVID-19 pandemic. A recent study in Korea reported similar findings that children in households with low and declining income engaged in more screen time than those in high-income households.^38^ Digital devices can be used not only in the educational field but also as a tool for social skills and playing games. An explanation might be that children’s screen time may be associated with parenting style, where the higher the family income, the better control over time spent using digital devices during the pandemic.^38^ Consistent with a previous cross-sectional study by Yam et al,^2^ girls had less myopia and a lower rate of myopia than boys (Table 4), which may be associated with the more time that boys spent on computers and electronic devices (Table 3).

Given the strong association between early-onset myopia and development of high myopia,^39,40^ the doubled myopia incidence found in younger children in this study causes concern about future rate increases in myopia. The lifestyle changes, including reduced time spent outdoors, increased screen time, and increased near-work time, persisted beyond the lift of pandemic restrictions and might increase the risk of prolonged progression of myopia. Notably, children from low-income households were exposed to longer screen time and, thus, may have been at a higher risk of myopic shift during the COVID-19 pandemic. Recommendations to mitigate these risk factors should target younger children and children from a lower socioeconomic background.

### Limitations

This study had several limitations. First, our results do not represent the effect of the COVID-19 pandemic on other parts of the world where social distancing, home quarantine, and school closure policies were different. Second, our participants were Chinese, so the generalizability of the study results as they would pertain to other racial and ethnic populations may be limited. Third, the lifestyles, including time spent outdoors and screen time, were obtained from questionnaires, which were subject to recall and reporting biases. Fourth, this study evaluated myopia trends in schoolchildren from 2015 to 2021. The pandemic is still ongoing, and a longer follow-up after the pandemic may yield a more complete picture.

## Conclusions

This cross-sectional study found that a high prevalence of myopia has persisted in young Chinese schoolchildren in Hong Kong, even after COVID-19 confinement measures were lifted. Children’s lifestyle was significantly changed and did not completely return to pre–COVID-19 levels. Younger children and those from families with a low household income were at a higher risk of myopia development during the pandemic, suggesting that collective efforts for myopia control should be advocated for these groups.

## Notes

Supplement 1. **eMethods.** Definition and Calculation for Outdoor Time, Screen Time, and Total Near-Work Time **eTable 1.** Association of Outdoor Time, Near-Work Time, Screen Time, and Diopter-Hours With Myopia Prevalence, Spherical Equivalent Refraction, and Axial Length in Children **eTable 2.** Myopia Prevalence, Spherical Equivalent Refraction, and Axial Length Before and During the COVID-19 Pandemic **eTable 3.** Outdoor Time, Near-Work Time, Screen Time, and Diopter-Hours Before and During the COVID-19 Pandemic **eTable 4.** Association of Outdoor Time, Near-Work Time, Screen Time, and Diopter-Hours With Myopia Prevalence, Spherical Equivalent Refraction, and Axial Length in Children With Low Family Income or High Family Income **eTable 5.** Association of Outdoor Time, Near Work Time, Screen Time, and Diopter-Hour with Myopia Prevalence, Spherical Equivalent, and Axial Length in Children Without Myopic Parents or Both Myopic Parents

Supplement 2.
Data Sharing Statement
