# Supplementary material for: Prevalence of Myopia in Children Before, During, and After COVID-19 Restrictions in Hong Kong
Source: JAMA Netw Open. 2023 Mar 22;6(3):e234080. doi: 10.1001/jamanetworkopen.2023.4080 (PMC10034576; doi:10.1001/jamanetworkopen.2023.4080)
Supplement: Supplement 1. — eMethods. Definition and Calculation for Outdoor Time, Screen Time, and Total Near-Work Time eTable 1. Association of Outdoor Time, Near-Work Time, Screen Time, and Diopter-Hours With Myopia Prevalence, Spherical Equivalent Refraction, and Axial Length in Children eTable 2. Myopia Prevalence, Spherical Equivalent Refraction, and Axial Length Before and During the COVID-19 Pandemic eTable 3. Outdoor Time, Near-Work Time, Screen Time, and Diopter-Hours Before and During the COVID-19 Pandemic eTable 4. Association of Outdoor Time, Near-Work Time, Screen Time, and Diopter-Hours With Myopia Prevalence, Spherical Equivalent Refraction, and Axial Length in Children With Low Family Income or High Family Income eTable 5. Association of Outdoor Time, Near Work Time, Screen Time, and Diopter-Hour with Myopia Prevalence, Spherical Equivalent, and Axial Length in Children Without Myopic Parents or Both Myopic Parents [file jamanetwopen-e234080-s001.pdf]

Supplemental Online Content

Zhang XJ, Zhang Y, Kam KW, et al. Prevalence of myopia in children before, during, and after COVID-19 restrictions in Hong Kong. *JAMA Netw Open*. 2023;6(3):e234080. doi:10.1001/jamanetworkopen.2023.4080

eMethods. Definition and Calculation for Outdoor Time, Screen Time, and Total Near-Work Time

eTable 1. Association of Outdoor Time, Near-Work Time, Screen Time, and Diopter-Hours With Myopia Prevalence, Spherical Equivalent Refraction, and Axial Length in Children

eTable 2. Myopia Prevalence, Spherical Equivalent Refraction, and Axial Length Before and During the COVID-19 Pandemic

eTable 3. Outdoor Time, Near-Work Time, Screen Time, and Diopter-Hours Before and During the COVID-19 Pandemic

eTable 4. Association of Outdoor Time, Near-Work Time, Screen Time, and Diopter-Hours With Myopia Prevalence, Spherical Equivalent Refraction, and Axial Length in Children With Low Family Income or High Family Income

eTable 5. Association of Outdoor Time, Near Work Time, Screen Time, and Diopter-Hour with Myopia Prevalence, Spherical Equivalent, and Axial Length in Children Without Myopic Parents or Both Myopic Parents

This supplemental material has been provided by the authors to give readers additional information about their work.

© 2023 Zhang XJ et al. *JAMA Network Open.*

eMethods. Definition and Calculation for Outdoor Time, Screen Time, and Total Near-Work Time

Categories	Items
**Outdoor time**	
	Outdoor for sports
	Outdoor for leisure [a]
**Reading and writing time**	
	Doing paper homework
	Reading and Writing
	Drawing and coloring
**Screen time**	
	Watching TV
	Using computer
	Electronic game / using smartphone or tablet PC for leisure
	Using smartphone or tablet PC for learning
**Total near work time**	
	Reading and writing time
	Screen time without time for watch TV, VCDs or online videos

[a] Include walking, leisure bike riding, playing in the park and picnicking.
Average number of daily hours=(weekday daytime hours × 5 + weekend daytime hours × 2) ÷ 7**.**
Diopter-hours = (study hours + leisure reading hours) × 3 + video game or home computer work hours × 2 + television hours × 1.

© 2023 Zhang XJ et al. *JAMA Network Open.*

eTable 1. Association of Outdoor Time, Near-Work Time, Screen Time, and Diopter-Hours With Myopia Prevalence, Spherical Equivalent Refraction, and Axial Length in Children

	Myopia prevalence				Spherical equivalent, D				Axial length, mm			
	Univariate		Multivariable		Univariate		Multivariable		Univariate		Multivariable	
	Exp (β) (95% CI)	P value	Exp (β) (95% CI)	P value	β (SE)	P value	β (SE)	P value	β (SE)	P value	β (SE)	P value
Outdoor time [a]	0.86 (0.80-0.92)	<0.001	0.88 (0.82-0.96)	0.002 [c]	0.08 (0.02)	0.001	0.05 (0.02)	0.04	0.00 (0.01)	0.93	-0.01 (0.01)	0.55
Near work time [b]	1.08 (1.06-1.11)	<0.001	1.04 (1.01-1.07)	0.004 [c]	-0.05 (0.01)	<0.001	-0.01 (0.01)	0.21	0.04 (0.01)	<0.001	0.01 (0.01)	0.09
Screen time [b]	1.03 (1.00-1.06)	0.08	1.00 (0.96-1.03)	0.77	-0.01 (0.01)	0.17	0.01 (0.01)	0.39	0.03 (0.01)	<0.001	0.00 (0.01)	0.51
Diopter-hour [b]	1.03 (1.02-1.04)	<0.001	1.01 (1.00-1.02)	0.009 [c]	-0.02 (0.00)	<0.001	0.00 (0.00)	0.28	0.01 (0.00)	<0.001	0.00 (0.00)	0.22

Abbreviation: Exp (β), odds ratio; CI, confidence interval; D, diopter; mm, millimeter; β, Beta coefficient; SE, standard error.

[a] Statistical value for outdoor time, the model was adjusted for age, sex, family income, parental myopia, and diopter-hour.

[b] Statistical values for near work time, screen time and diopter-hour were generated from separate multivariable regression models due to correlation between them, which adjusted for age, sex, family income, parental myopia, and outdoor time.

[c] There was no significant interaction effect between outdoor time and near work time or diopter-hour on myopia prevalence.

© 2023 Zhang XJ et al. *JAMA Network Open.*

eTable 2. Myopia Prevalence, Spherical Equivalent Refraction, and Axial Length Before and During the COVID-19 Pandemic

	Myopia prevalence				
	Before COVID-19 pandemic	During COVID-19 pandemic	Exp (β) (95% CI)	Adjusted Exp (β) (95% CI) [a]	*P* value [a]
All subjects	23.8%	33.9%	1.65 (1.53 to 1.78)	1.47 (1.36 to 1.59)	<0.001
Age, years					
6	13.9%	22.8%	1.83 (1.57 to 2.14)	1.70 (1.45 to 1.99)	<0.001
7	25.8%	32.8%	1.63 (1.44 to 1.84)	1.55 (1.38 to 1.75)	<0.001
8	37.3%	45.2%	1.39 (1.22 to 1.58)	1.39 (1.22 to 1.58)	<0.001
Sex					
Male	24.3%	34.8%	1.66 (1.50 to 1.84)	1.48 (1.33 to 1.64)	<0.001
Female	23.2%	33.0%	1.63 (1.46 to 1.82)	1.47 (1.31 to 1.65)	<0.001
No. of parental myopia					
0	15.3%	26.2%	1.96 (1.66 to 2.30)	1.77 (1.50 to 2.08)	<0.001
1	20.5%	30.3%	1.69 (1.46 to 1.95)	1.47 (1.26 to 1.70)	<0.001
2	30.2%	36.4%	1.32 (1.14 to 1.53)	1.13 (0.97 to 1.32)	0.11
Family income, HK$ per month					
<25,000	22.1%	33.3%	1.59 (1.36 to 1.85)	1.42 (1.21 to 1.65)	<0.001
25,000-49,999	23.7%	33.8%	1.52 (1.30 to 1.77)	1.28 (1.09 to 1.51)	0.002
≥50,000	25.9%	35.0%	1.48 (1.27 to 1.72)	1.25 (1.06 to 1.46)	0.01
	Spherical Equivalent, D				
	Before COVID-19 pandemic	During COVID-19 pandemic	Mean difference	Adjusted mean difference [b]	*P* value [b]
All subjects	0.28 (1.47)	-0.02 (1.51)	-0.30 (-0.35 to -0.25)	-0.20 (-0.25 to -0.15)	<0.001
Age, years					
6	0.64 (1.21)	0.35 (1.37)	-0.29 (-0.37 to -0.21)	-0.25 (-0.33 to -0.17)	<0.001
7	0.21 (1.48)	0.01 (1.42)	-0.21 (-0.29 to -0.12)	-0.21 (-0.29 to -0.12)	<0.001
8	-0.23 (1.66)	-0.38 (1.64)	-0.15 (-0.26 to -0.05)	-0.15 (-0.25 to -0.04)	0.006
Sex					
Male	0.24 (1.48)	-0.08 (1.50)	-0.32 (-0.39 to -0.25)	-0.22 (-0.29 to -0.14)	<0.001
Female	0.32 (1.51)	0.04 (1.51)	-0.28 (-0.35 to -0.20)	-0.18 (-0.25 to -0.11)	<0.001
No. of parental myopia					
0	0.51 (1.40)	0.14 (1.49)	-0.37 (-0.48 to -0.27)	-0.30 (-0.40 to -0.19)	<0.001

© 2023 Zhang XJ et al. *JAMA Network Open.*

1	0.28 (1.50)	0.00 (1.47)	-0.28 (-0.37 to -0.18)	-0.22 (-0.32 to -0.12)	<0.001
2	-0.12 (1.63)	-0.21 (1.60)	-0.09 (-0.21 to 0.03)	-0.04 (-0.16 to 0.07)	0.50
Family income, HK$ per month					
<25,000	0.32 (1.51)	0.01 (1.56)	-0.32 (-0.50 to -0.25)	-0.27 (-0.37 to -0.16)	<0.001
25,000-49,999	0.28 (1.57)	-0.02 (1.53)	-0.30 (-0.41 to -0.20)	-0.15 (-0.26 to -0.04)	0.01
≥50,000	0.23 (1.56)	-0.05 (1.51)	-0.28 (-0.39 to -0.17)	-0.10 (-0.21 to 0.01)	0.09
	Axial Length, mm				
	Before COVID-19 pandemic	During COVID-19 pandemic	Mean difference	Adjusted mean difference [b]	*P* value [b]
All subjects	23.00 (0.90)	23.17 (0.93)	0.16 (0.13 to 0.19)	0.07 (0.05 to 0.10)	<0.001
Age, years					
6	22.71 (0.78)	22.83 (0.89)	0.12 (0.07 to 0.17)	0.09 (0.04 to 0.14)	<0.001
7	23.08 (0.90)	23.17 (0.87)	0.09 (0.04 to 0.14)	0.09 (0.04 to 0.14)	<0.001
8	23.38 (0.93)	23.46 (0.94)	0.08 (0.03 to 0.14)	0.08 (0.02 to 0.13)	0.009
Sex					
Male	23.26 (0.87)	23.44 (0.90)	0.18 (0.14 to 0.22)	0.10 (0.06 to 0.14)	<0.001
Female	22.72 (0.85)	22.86 (0.88)	0.14 (0.10 to 0.18)	0.07 (0.03 to 0.11)	0.001
No. of parental myopia					
0	22.90 (0.87)	23.10 (0.91)	0.19 (0.13 to 0.26)	0.13 (0.07 to 0.19)	<0.001
1	23.00 (0.91)	23.18 (0.92)	0.18 (0.11 to 0.24)	0.08 (0.02 to 0.13)	0.009
2	23.16 (0.96)	23.29 (0.95)	0.13 (0.06 to 0.20)	0.00 (-0.06 to 0.06)	0.99
Family income, HK$ per month					
<25,000	22.93 (0.90)	23.12 (0.93)	0.19 (0.12 to 0.26)	0.09 (0.02 to 0.15)	0.006
25,000-49,999	23.01 (0.93)	23.18 (0.94)	0.17 (0.10 to 0.24)	0.07 (0.01 to 0.14)	0.03
≥50,000	23.05 (0.94)	23.19 (0.91)	0.15 (0.08 to 0.22)	0.02 (-0.05 to 0.08)	0.59

Abbreviation: Exp (β), odds ratio; CI, confidence interval; D, diopter; mm, millimeter.

[a] Adjusted odds ratio and *P* value were generated by the logistic regression with the adjustment of age and sex.

[b] Adjusted mean difference and *P* value were generated by the generalized estimating equation model with the adjustment of age and sex.

© 2023 Zhang XJ et al. *JAMA Network Open.*

eTable 3. Outdoor Time, Near-Work Time, Screen Time, and Diopter-Hours Before and During the COVID-19 Pandemic

	Outdoor time, hours per day		Near work time, hours per day		Screen time, hours per day		Diopter-hours per day	
	Before COVID-19 pandemic	During COVID-19 pandemic	Before COVID-19 pandemic	During COVID-19 pandemic	Before COVID-19 pandemic	During COVID-19 pandemic	Before COVID-19 pandemic	During COVID-19 pandemic
All subjects	1.43 (0.61)	1.16 (0.53)	3.33 (1.36)	4.91 (1.89)	2.03 (1.21)	3.11 (1.73)	9.82 (3.74)	14.17 (5.02)
Age, years								
6	1.45 (0.62)	1.14 (0.54)	3.10 (1.27)	4.77 (1.87)	1.92 (1.18)	3.04 (1.73)	9.22 (3.50)	13.75 (4.91)
7	1.43 (0.60)	1.13 (0.50)	3.43 (1.37)	4.96 (1.78)	2.03 (1.17)	3.08 (1.60)	10.07 (3.82)	14.29 (4.75)
8	1.41 (0.60)	1.22 (0.54)	3.58 (1.41)	4.98 (2.04)	2.21 (1.29)	3.21 (1.90)	10.45 (3.88)	14.33 (5.44)
Sex								
Male	1.47 (0.61)	1.18 (0.52)	3.31 (1.35)	4.86 (1.92)	2.07 (1.22)	3.17 (1.81)	9.69 (3.72)	13.96 (5.03)
Female	1.39 (0.60)	1.14 (0.54)	3.36 (1.36)	4.98 (1.86)	1.98 (1.20)	3.04 (1.64)	9.95 (3.76)	14.40 (5.01)
No. of parental myopia								
0	1.47 (0.66)	1.19 (0.58)	3.37 (1.37)	5.03 (2.01)	2.14 (1.27)	3.30 (1.89)	9.82 (3.73)	14.41 (5.31)
1	1.44 (0.62)	1.17 (0.53)	3.37 (1.36)	4.89 (1.87)	2.06 (1.20)	3.10 (1.71)	9.93 (3.77)	14.14 (4.97)
2	1.40 (0.56)	1.13 (0.48)	3.27 (1.34)	4.86 (1.82)	1.92 (1.17)	3.00 (1.64)	9.69 (3.72)	14.03 (4.87)
Family income, HK$ per month								
<25,000	1.45 (0.66)	1.18 (0.59)	3.41 (1.39)	5.16 (2.05)	2.17 (1.28)	3.44 (1.97)	9.96 (3.77)	14.71 (5.39)
25,000-49,999	1.42 (0.60)	1.16 (0.52)	3.37 (1.35)	4.76 (1.78)	2.10 (1.21)	3.06 (1.60)	9.96 (3.77)	13.78 (4.73)
≥50,000	1.43 (0.53)	1.15 (0.48)	3.18 (1.27)	4.83 (1.85)	1.78 (1.07)	2.90 (1.61)	9.48 (3.58)	14.00 (4.99)

© 2023 Zhang XJ et al. *JAMA Network Open.*

eTable 4. Association of Outdoor Time, Near-Work Time, Screen Time, and Diopter-Hours With Myopia Prevalence, Spherical Equivalent Refraction, and Axial Length in Children With Low Family Income or High Family Income

Children with low family income[a] (n=8257)	Myopia prevalence				Spherical equivalent, D				Axial length, mm			
	Univariate		Multivariable		Univariate		Multivariable		Univariate		Multivariable	
	Exp (β) (95% CI)	P value	Exp (β) (95% CI)	P value	β (SE)	P value	β (SE)	P value	β (SE)	P value	β (SE)	P value
Outdoor time[b]	0.88 (0.79-0.98)	0.02	0.88 (0.78-0.99)	0.04[d]	0.08 (0.03)	0.01	0.07 (0.04)	0.04	0.00 (0.02)	0.76	0.00 (0.02)	0.95
Near work time[c]	1.08 (1.04-1.13)	<0.001	1.05 (1.00-1.09)	0.04[c]	-0.05 (0.02)	0.002	-0.01 (0.01)	0.33	0.04 (0.01)	<0.001	0.01 (0.01)	0.29
Screen time[c]	1.05 (1.00-1.09)	0.04	1.02 (0.97-1.07)	0.52	-0.03 (0.01)	0.08	0.00 (0.01)	0.88	0.03 (0.01)	0.003	0.00 (0.01)	0.93
Diopter-hour[c]	1.03 (1.02-1.05)	<0.001	1.02 (1.00-1.04)	0.03[c]	-0.02 (0.01)	0.003	-0.01 (0.01)	0.29	0.01 (0.00)	0.001	0.00 (0.00)	0.40
Children with high family income[a] (n=5916)	Myopia prevalence				Spherical equivalent, D				Axial length, mm			
	Univariate		Multivariable		Univariate		Multivariable		Univariate		Multivariable	
	Exp (β) (95% CI)	P value	Exp (β) (95% CI)	P value	β (SE)	P value	β (SE)	P value	β (SE)	P value	β (SE)	P value
Outdoor time[b]	0.86 (0.75-0.98)	0.03	0.84 (0.72-0.98)	0.02	0.05 (0.05)	0.32	0.07 (0.05)	0.17	-0.01 (0.03)	0.77	-0.04 (0.03)	0.17
Near work time[c]	1.09 (1.04-1.14)	<0.001	1.04 (0.99-1.09)	0.15	-0.04 (0.02)	0.01	0.00 (0.02)	0.99	0.03 (0.01)	0.001	0.01 (0.01)	0.61
Screen time[c]	1.00 (0.95-1.06)	0.87	1.01 (0.94-1.07)	0.65	-0.01 (0.02)	0.60	0.00 (0.02)	0.76	0.02 (0.01)	0.17	0.00 (0.01)	0.87
Diopter-hour[c]	1.03 (1.01-1.04)	0.001	1.01 (0.99-1.03)	0.27	-0.01 (0.01)	0.03	0.00 (0.01)	0.83	0.01 (0.00)	0.01	0.00 (0.00)	0.99

Abbreviation: Exp (β), odds ratio; CI, confidence interval; D, diopter; mm, millimeter; β, Beta coefficient; SE, standard error.

[a] Low family income was defined as family income less than HK $25,000 per month and high family income was defined as family income equal or greater than HK $50,000 per month.

[b] Statistical value for outdoor time, the model was adjusted for age, sex, family income, parental myopia, and diopter-hour.

[c] Statistical values for near work time, screen time and diopter-hour were generated from separate multivariable regression models due to correlation between them, which adjusted for age, sex, family income, parental myopia, and outdoor time.

[d] There was no significant interaction effect between outdoor time and near work time or diopter-hour on myopia prevalence.

© 2023 Zhang XJ et al. *JAMA Network Open.*

eTable 5. Association of Outdoor Time, Near Work Time, Screen Time, and Diopter-hour with Myopia Prevalence, Spherical Equivalent and Axial Length in Children without Myopic Parents or Both Myopic Parents

Children without myopic parents (n=6406)	Myopia prevalence				Spherical equivalent, D				Axial length, mm			
	Univariate		Multivariable		Univariate		Multivariable		Univariate		Multivariable	
	OR (95% CI)	P value	OR (95% CI)	P value	β (SE)	P value	β (SE)	P value	β (SE)	P value	β (SE)	P value
Outdoor time [a]	0.85 (0.73-0.99)	0.04	0.86 (0.76-0.98)	0.02	0.12 (0.04)	0.009	0.11 (0.04)	0.01	0.00 (0.03)	0.73	-0.01 (0.03)	0.78
Near work time [b]	1.08 (1.02-1.14)	0.006	1.04 (0.98-1.10)	0.16	-0.04 (0.02)	0.03	-0.02 (0.02)	0.39	0.04 (0.01)	0.001	0.02 (0.01)	0.04
Screen time [b]	1.05 (0.99-1.12)	0.08	1.02 (0.96-1.09)	0.46	-0.03 (0.02)	0.06	-0.01 (0.02)	0.43	0.04 (0.01)	0.003	0.02 (0.01)	0.16
Diopter-hour [b]	1.03 (1.01-1.05)	0.009	1.02 (0.99-1.04)	0.15	-0.01 (0.01)	0.04	-0.01 (0.01)	0.42	0.01 (0.00)	0.003	0.01 (0.00)	0.08
Children with both myopic parents (n=6527)	Myopia prevalence				Spherical equivalent, D				Axial length, mm			
	Univariate		Multivariable		Univariate		Multivariable		Univariate		Multivariable	
	OR (95% CI)	P value	OR (95% CI)	P value	β (SE)	P value	β (SE)	P value	β (SE)	P value	β (SE)	P value
Outdoor time [a]	0.87 (0.77-0.97)	0.02	0.89 (0.75-1.05)	0.16	0.05 (0.04)	0.25	0.04 (0.05)	0.34	-0.01 (0.03)	0.85	-0.03 (0.02)	0.18
Near work time [b]	1.07 (1.03-1.11)	0.001	1.01 (0.97-1.05)	0.71	-0.05 (0.02)	0.01	-0.01 (0.02)	0.39	0.04 (0.01)	<0.001	0.00 (0.01)	0.90
Screen time [b]	1.01 (0.96-1.06)	0.64	1.01 (0.96-1.06)	0.83	-0.01 (0.02)	0.61	-0.01 (0.02)	0.89	0.01 (0.01)	0.25	0.01 (0.01)	0.42
Diopter-hour [b]	1.02 (1.01-1.04)	0.002	1.00 (0.99-1.02)	0.77	-0.02 (0.01)	0.02	-0.01 (0.01)	0.37	0.01 (0.00)	0.003	0.00 (0.00)	0.85

Abbreviation: Exp (β), odds ratio; CI, confidence interval; D, diopter; mm, millimeter; Beta (β), Beta coefficient; SE, standard error.

[a] Statistical value for outdoor time, the model was adjusted for age, sex, family income, parental myopia, and diopter-hour.

[b] Statistical values for near work time, screen time and diopter-hour were generated from separate multivariable regression models due to correlation between them, which adjusted for age, sex, family income, parental myopia, and outdoor time.

© 2023 Zhang XJ et al. *JAMA Network Open.*
