# Supplementary material for: Prevalence of Myopia in Children Before, During, and After COVID-19 Restrictions in Hong Kong
Source: JAMA Netw Open. 2023 Mar 22;6(3):e234080. doi: 10.1001/jamanetworkopen.2023.4080 (PMC10034576; doi:10.1001/jamanetworkopen.2023.4080)
Supplement: Supplement 2. — Data Sharing Statement [file jamanetwopen-e234080-s002.pdf]

Data Sharing Statement

Zhang. Prevalence of Myopia in Children Before, During, and After COVID-19 Restrictions in Hong Kong. *JAMA Netw Open*. Published March 22, 2023.
doi:10.1001/jamanetworkopen.2023.4080

Data
Data available: No
